# Epileptic arousals: A neglected clinical entity

**DOI:** 10.1002/epi.70442

**Published:** 2026-08-19

**Authors:** Lea Fisel, Nóra Pető, Sotirios Kalousios, Nicolas Zabler, Armin Brandt, Peter C. Reinacher, Dieter Riemann, Mohammad F. Khazali, Andreas Schulze‐Bonhage

**Affiliations:** ^1^ Epilepsy Center Faculty of Medicine, Medical Center ‐ University of Freiburg, Member of the ERN EpiCARE Freiburg Germany; ^2^ Department of Neurology Central Hospital of Northern Pest ‐ Military Hospital Budapest Hungary; ^3^ BrainLinks‐BrainTools Freiburg Germany; ^4^ Department of Stereotactic and Functional Neurosurgery Faculty of Medicine, Medical Center ‐ University of Freiburg Freiburg Germany; ^5^ Department of Psychiatry and Psychotherapy Medical Center ‐ University of Freiburg, Faculty of Medicine Freiburg Germany; ^6^ German Sleep Society (DGSM e.V.) Jena Germany; ^7^ Department of Epileptology University Hospital Bonn Bonn Germany

**Keywords:** autonomic nervous system, focal epilepsy, heart rate, intracranial electroencephalography, sleep‐related seizures

## Abstract

**Objective:**

Epileptic arousals (EAs) are seizures characterized solely by arousal from sleep. EAs are unrecognized in current seizure classifications. Their subtle semiology and inconsistently detectable ictal activity in scalp electroencephalography (EEG) complicate differentiation from physiological arousals (PAs). This study characterizes EAs using simultaneous intracranial electroencephalography (iEEG) and scalp EEG with electrocardiography (ECG), exploring their neural and autonomic dynamics.

**Methods:**

We retrospectively analyzed presurgical video‐EEG monitoring data of 20 focal epilepsy patients (2010–2023). EAs were identified visually. Their seizure‐onset zones (SOZs) and seizure‐propagation zones (SPZs) were defined in Montreal Neurological Institute (MNI) space and compared to other seizure types. The detectability of ictal activity of EAs in scalp EEG was evaluated. Associations with SOZs/SPZs were assessed using logistic regression. Heart rate dynamics were compared between EAs and PAs evaluating distribution modality, variability, and receiver operating characteristic (ROC) analysis.

**Results:**

Among 507 seizures, 133 (26.2%) were EAs. SOZs of EAs partially overlapped with other seizure types (Jaccard similarity index 0.47). Two‐thirds (66.9%) of EAs were undetectable by scalp EEG. Detectability increased with broader maximal SPZs (odds ratio [OR] = 3.16; ROC‐AUC [area under the curve] = 0.891). Heart rate increased during both EAs and PAs (*p* < 0.001), yet modulation was greater during EAs (*p* = 0.007, ROC‐AUC = 0.995) and showed a multimodal distribution (*p* < 0.001).

**Significance:**

EAs are common, yet underrecognized. They arise from partially distinct networks and may exhibit a specific autonomic signature, supporting their recognition as a separate seizure type. Reliance on scalp EEG alone risks missing patients with a high EA burden. Undetected EAs can perpetuate epileptic activity and sleep fragmentation.

**Key points**

- Epileptic arousals (EAs) are seizures with arousal from sleep as only semiology.
- Combined intracranial and scalp electroencephalography (EEG) with electrocardiography (ECG) provide a rare opportunity to study EAs.
- EAs arise from partially distinct networks compared to other seizure types.
- EAs exhibit a specific autonomic signature compared to physiological arousals.
- Ictal activity of most EAs is not detectable in scalp EEG, making differentiation from physiological arousal, correct diagnosis, and treatment challenging.

## 1 INTRODUCTION

### 1.1 Physiological and pathophysiological arousals in sleep

Arousals are transient shifts in brain state toward wakefulness, involving hierarchical activation of subcortical, cortical, autonomic, and somatomotoric components. Physiological arousals (PAs) occur spontaneously or induced by exogenous (e.g., noise) or endogenous stimuli (e.g., bladder distension). Arousals fulfill essential regulatory and protective functions: facilitating sleep reversibility, maintaining environmental monitoring, and supporting homeostatic, restorative, and plasticity‐promoting processes.^1^

Conversely, PAs arise from abnormal or excessive stimuli or brain activity that disrupts sleep continuity and are often disease related. Triggers include intrinsic sleep disorders (e.g., obstructive sleep apnea, parasomnias), somatic or psychiatric conditions (e.g., pain or affective disorders), and external factors (e.g., medication, substance abuse). The influence of pathophysiological stimuli on arousals remains poorly defined, especially in neurological diseases such as epilepsy.

### 1.2 Sleep, arousal and epilepsy

Imbalance between arousal‐ and sleep‐promoting systems leads to sleep instability and fragmentation, observed in sleep and arousal disorders^2^ and epilepsy.^3^ Epilepsy, one of the most common neurological disorders worldwide, is characterized by transient episodes of pathological, excessive, or hypersynchronous neuronal activity—epileptic seizures.^4^ Their clinical manifestations vary depending on the brain regions involved in seizure onset and propagation.^5^

The relationship between sleep and epilepsy is bidirectional: Sleep modulates ictal and interictal activity.^6^ Conversely, ictal activity and interictal activity^7^ disrupt sleep architecture and quality. Less is known about the reciprocal influence of arousal and epilepsy, particularly regarding the underrecognized phenomenon of epileptic arousals (EAs).

### 1.3 Epileptic arousals: Concept and terminology

Epileptic arousals (or EAs) are seizures with an arousal/awakening from sleep as the sole semiological feature. Ictal activity induces the arousal, first described by Malow and Varma^8^ in a single case report and later in a cohort of 14 patients.^9^ EAs received little scientific and clinical attention. They are neither included in the current International League Against Epilepsy (ILAE) classification of seizure types^10^ nor recent reviews about sleep and epilepsy.^11^

Consequently, EAs have been described mainly in case reports and small retrospective studies. A standardized definition and characterization are lacking, and terminology is heterogeneous with terms like “hypnopompic seizures,”^12^, ^13^ “ictal arousals,”^14^ “seizures with arousing effects,”^7^, ^15^ and “epileptic arousals.”^16^, ^17^ It remains unclear whether these terms refer exclusively to EAs or to a broader range of seizure types in which arousals are not the sole semiology.

In this study, we propose a formal definition of EAs to enable consistent classification (see 2.1).

### 1.4 Challenges in detecting epileptic arousals

EAs are subtle. Furthermore, scalp electroencephalography (EEG) has limited sensitivity in capturing their temporal^8^, ^9^ and spatial features.^13^ Its sensitivity to detect associated ictal activity remains unclear. Consequently, in non‐invasive settings using only scalp EEG it is challenging to determine whether an arousal represents an epileptic seizure (EA) or merely a physiological element of sleep (PA).

To date, reliable identification of EA requires simultaneous recording of intracranial EEG (iEEG) and scalp EEG obtained during presurgical evaluation in patients with focal epilepsy.^8^, ^9^, ^13^

### 1.5 Study objectives and hypotheses

This study systematically characterizes EA as an underrecognized seizure type, proposing the term “*epileptic arousal.”* Using iEEG as ground truth to identify ictal activity and scalp EEG as gold standard for sleep and arousal evaluation, we leverage their simultaneous acquisition as a unique opportunity to study EAs.

We hypothesize that.

(1) EAs represent a distinct seizure type;
(2) a substantial proportion of EAs cannot be reliably detected using scalp EEG alone;
(3) the detectability of ictal activity in scalp EEG is associated with the localization and spatial extent of seizure activity in iEEG; and
(4) although both EAs and PAs modulate heart rate (HR), EAs exhibit distinct HR dynamics.

## 2 MATERIALS AND METHODS

### 2.1 Definition of epileptic arousals

We define EAs as epileptic seizures in which micro‐ or macro‐arousal constitutes the sole semiological feature. Arousal is not represented as a seizure descriptor in the current ILAE classification of seizure types.^10^

EAs may involve arousal‐associated phenomena of cortical activation, autonomic responses, and motor activity (e.g., waking up, eye opening, increasing heart rate, moving in bed; Figure 1).

**FIGURE 1:**
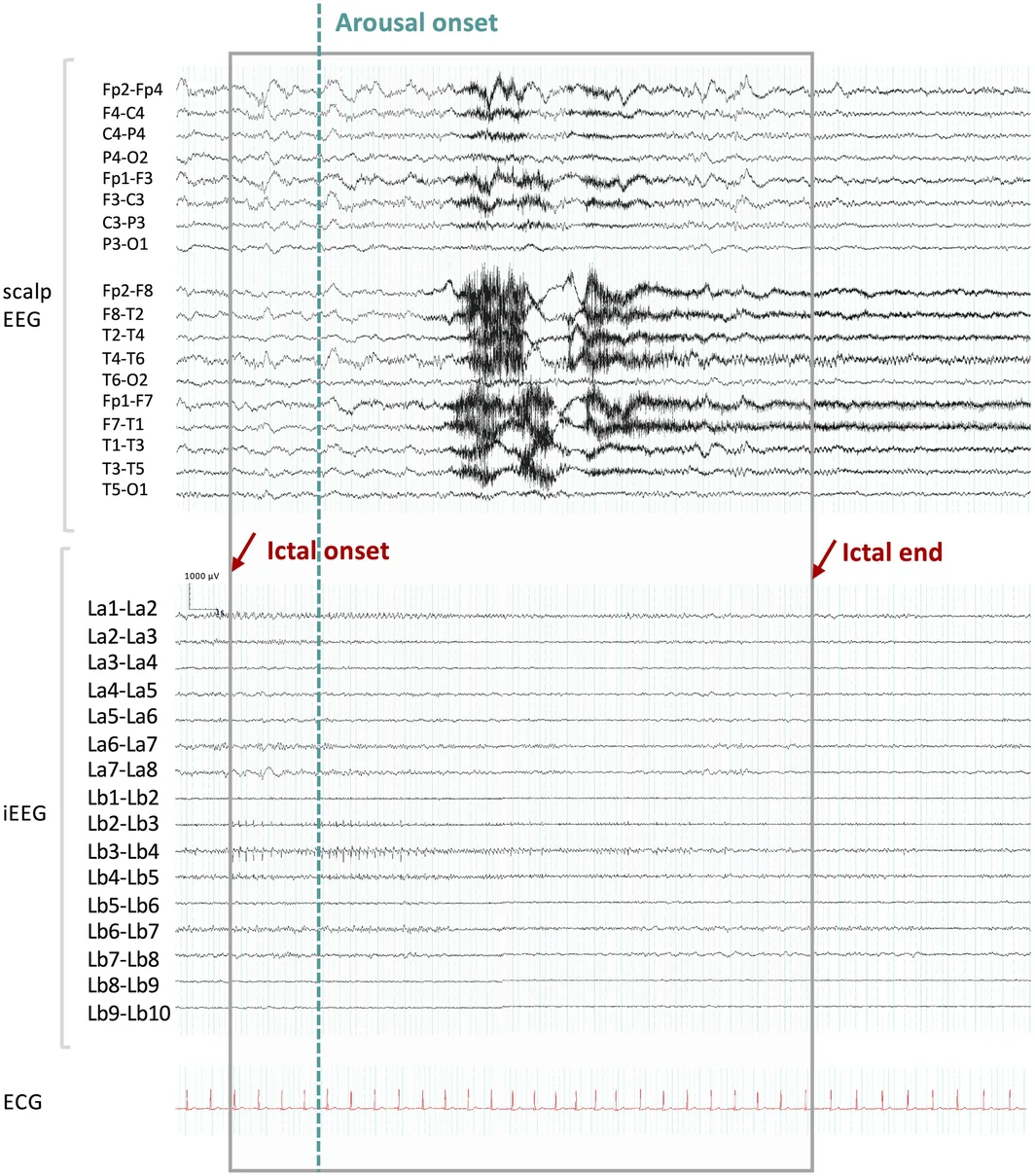
Epileptic arousal in simultaneous intracranial and scalp EEG. The ictal activity as seen in intracranial recordings from the precentral gyrus iEEG contact (Lb3‐4) induces a fast‐type arousal during N2 sleep, which subsequently becomes apparent on scalp EEG (dashed line). On scalp EEG, there is no ictal activity detectable. Consequently, using scalp EEG alone, the epileptic arousal would be misclassified as physiological arousal. ECG, electrocardiography; EEG, electroencephalography; iEEG, intracranial EEG.

Beyond that, EAs particularly do not include any of the phenomena listed as descriptors in the current ILAE classification.

Because arousals frequently accompany various seizure types, EAs require that the arousal represents the predominant semiology, without transitions into other seizure types. We define those as “other seizure types with arousing effect” or “other seizures accompanied by arousals” (e.g., focal to bilateral tonic–clonic seizures during sleep that result in arousals). Conversely, subclinical seizures are characterized by ictal activity with the absence of objective or subjective clinical manifestations. In contrast, PAs occur in the absence of ictal activity (Figure S1).

### 2.2 Data selection

We retrospectively screened patients undergoing presurgical diagnostics at the Epilepsy Center Freiburg (January 2010–January 2023) using a database query (epil*arous*) to identify reported EAs, a clinic‐intern descriptor routinely used in EEG annotations and medical reports. Inclusion criteria were: (i) patients with simultaneous iEEG, scalp EEG, one‐channel ECG, and synchronized audio‐visual recordings; (ii) ages of 5–99 years; (iii) patients with EAs occurring during sleep stages N2, N3 or rapid eye movement (REM) sleep, independently verified to meet predefined criteria of EAs (see Section 2.1). We excluded patients with comorbid sleep or arousal disorders. The study was conducted in accordance with the Declaration of Helsinki and approved by the Ethics Committee of the Albert‐Ludwigs‐Universität Freiburg (23‐1020‐S1‐retro).

### 2.3 Localization of intracranial electrodes

Depth electrodes (Ad‐Tech) were stereotactically implanted using a Leksell‐G frame (Elekta) according to individualized clinical hypotheses for epileptogenic focus localization.^18^ We included only stereo‐EEG recordings with depth electrodes. We excluded subdural electrodes to improve anatomic consistency across patients and facilitate precise three‐dimensional (3D) localization in Montreal Neurological Institute (MNI) space, a standardized brain coordinate system.^19^ To localize electrode contacts, post‐implantation magnetic resonance imaging (MRI) scans were processed, using a clinic‐internal pipeline for MNI transformation and skull‐stripping. We manually marked electrode contacts as regions of interest and extracted their MNI coordinates (MRIcron, version v1.0.20190902). We assigned anatomic locations using three complementary neuroanatomic atlases to improve robustness: Harvard–Oxford cortical and subcortical structural atlas,^20^ Johns Hopkins University (JHU)–MNI single subject atlas,^21^ and JHU White Matter tractography atlas.^22^ In case of discrepancies, we prioritized the atlas with higher spatial resolution. Finally, we reclassified anatomic regions into 49 broader categories to facilitate group‐level analysis.

### 2.4 EEG recording and assessment

After implantation of intracranial depth electrodes, scalp EEG was acquired after a median of 3 days (interquartile range [IQR] 0.25 [1–4]) using the international 10–20 system with additional T1 and T2 electrodes and maintained until explantation. Duration of simultaneous scalp EEG and iEEG recordings was a median of 7.5 nights (IQR 3.5 [2–15]). EEG signals were recorded at 2000 Hz, using Neuvo amplifiers and ProFusion software (version 7, Compumedics). Scalp EEG was reviewed using standard display setting (0.5 Hz high‐pass, 70 Hz low‐pass, 50 Hz notch, sensitivity 7 μV/mm, time base 10 s/page for epilepsy, and 30 s/page for sleep assessments), and, when necessary, under alternative clinically used filter and gain settings as part of routine EEG interpretation. In cases of uncertainty or discrepancy between raters, assessment required consensus among three authors (L.F., N.P., and A.S.B.) following joint review.

### 2.5 Ictal parameters in iEEG

We assessed whether the seizure‐onset zone (SOZ) was identifiable at the patient‐level, based on complete seizure type sampling and adequate iEEG spatial coverage. Independently, we determined the SOZ of each EA. Seizure onset was defined as time of the first distinct change in iEEG deviating from background activity and evolving into clear ictal activity. One or more channels exhibiting ictal activity at seizure onset, with a maximum temporal difference of <200 ms between channels, were visually identified, mapped to their corresponding anatomic locations (see Section 2.3), and designated as SOZ. We quantified the extent of SOZ by the number of involved electrode contacts and anatomic regions. For comparison, we obtained SOZ of non‐EA seizure types, including subclinical seizures, from clinical reports. We constructed a synoptic SOZ per patient encompassing all involved anatomic regions. Spatial overlap between the SOZ of EA and the synoptic SOZ was quantified as Jaccard similarity index. The maximum seizure‐propagation zone (max. SPZ) of EA was visually identified as iEEG channels exhibiting ictal activity at the time of maximum propagation, mapped to their corresponding anatomic locations, and quantified by the number of involved electrode contacts and anatomic regions. The duration of ictal iEEG discharges was determined based on visual inspection of the iEEG only.

### 2.6 Ictal parameters in scalp EEG

Scalp EEG was visually analyzed for each EA, and ictal activity was classified as detectable or undetectable based on the presence or absence of visually identifiable ictal patterns in scalp EEG. We evaluated detectability in scalp EEG within and across patients.

### 2.7 Analysis of the relationship between the localization and extent of ictal activity in iEEG and detectability of ictal activity in scalp EEG

Logistic regression analyses were used to assess whether the detectability of ictal activity in scalp EEG was associated with the localization and spatial extent of intracranial ictal activity. The dependent variable was scalp EEG detectability of ictal activity (detectable vs not detectable). Predictors were:

(a) proportion of EAs per patient with SOZ including the dorsolateral convexity;
(b) proportion of EAs per patient with max SPZ including the dorsolateral convexity; and
(c) median number of anatomic regions per patient involved in max SPZ.

Model performance was evaluated using receiver operating characteristic (ROC) analysis.

### 2.8 Sleep and arousal parameters

We visually scored sleep and arousal parameters in scalp EEG according to American Academy of Sleep Medicine (AASM) criteria.^23^ As full polysomnography, including electromyography (EMG) and electrooculography (EOG), is typically unavailable during pre‐surgical video‐EEG monitoring, we approximated REM sleep using surrogate markers like REM artifacts and absence of muscle artifacts. Because AASM criteria include only “fast” arousals and do not encompass the full spectrum of arousal types,^1^ we extended the classification by incorporating the cyclic alternating pattern (CAP) framework of sleep microstructure^24^ to also include “intermediate” and “slow” arousals (see supplementary material for definition). Arousal‐related frequency shifts of all types must last ≥3 s following ≥10 s of stable sleep. We defined arousal onset as the first distinct change in scalp EEG, or clinical onset (e.g., eye opening or motor reactions) if obscured with overlapping seizure patterns or muscle artifacts.

### 2.9 Matching of physiological arousals

For each patient and night with at least one EA in N2 sleep, we manually identified three PAs in N2, regardless of type, through targeted scalp EEG screening. Selection was restricted to the same night to minimize potentially confounding medical and environmental factors. The absence of ictal activity was verified in iEEG. PAs occurring within 1 min of each other were excluded to increase event independence.

### 2.10 Heart rate analysis

One‐channel ECG was recorded at 2000 Hz, with subclavicular and intercostal, or bilateral subclavicular electrode placement. We conducted HR analysis for both EAs and PAs during N2 sleep, to ensure comparability across sleep‐stage dependent autonomic activity. We excluded arousals during which clinical neurological assessments were performed, as such interventions may influence HR dynamics.

#### 2.10.1 Pre‐processing

ECG data were exported as 60‐s pre‐ and post‐seizure onset segments. Alignment to arousal onset required discarding the outermost 10 s of each segment, resulting in 50‐s pre‐ and post‐arousal segments. R‐peaks were detected using the Pan‐Tompkins algorithm^25^ and visually inspected to remove false positives. False negatives were not corrected. Instead, HR data with unreliable R‐peak detection or persistent artifacts were excluded. We calculated instantaneous HR and aligned it to the arousal onset in scalp EEG. Monotone cubic Hermite interpolation provided 0.1‐s precision. To reduce interpolation edge effects, the first 2 s of the pre‐arousal segment and the last 2 s of the post‐arousal segment were discarded, resulting in an effective analysis time of 48 s pre‐ and post‐arousal (Figure 2). To facilitate interindividual comparisons and adjust for confounding factors (e.g., sex, age, cardiovascular fitness), we normalized HR by dividing it by the highest value per patient. Baseline HR was adjusted by subtracting the median pre‐arousal HR from each patient's data.

**FIGURE 2:**
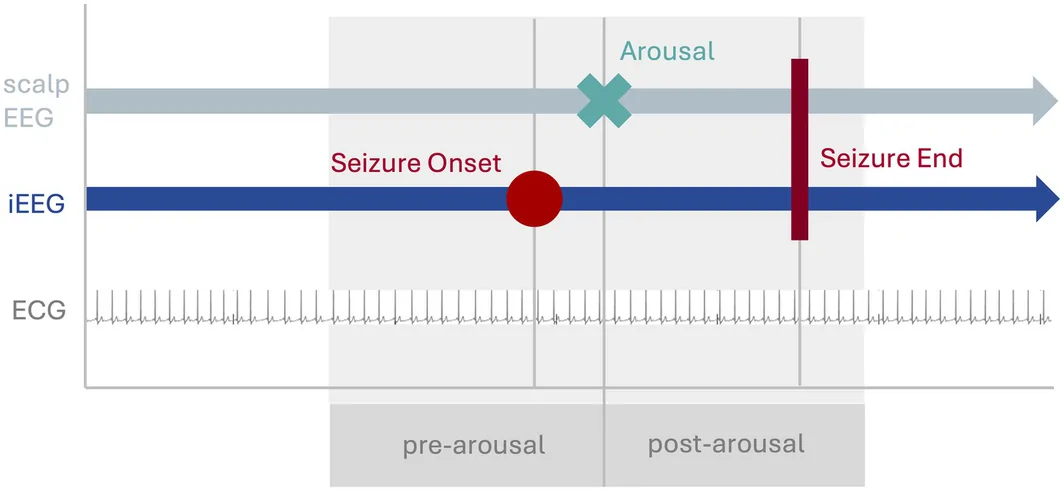
Schematic representation of peri‐arousal period of epileptic arousals. Seizure onset is detected in iEEG as ground truth. The ictal activity induces an arousal as seen on scalp EEG. Time windows pre‐ and post‐arousal were analyzed. ECG, electrocardiography; EEG, electroencephalography; iEEG, intracranial EEG.

#### 2.10.2 Statistical analysis

We performed quantitative analysis of median HR time series for EAs and PAs using the Wilcoxon rank‐sum test, based on median HR values computed per patient and timepoint. Pre‐ and post‐arousal phases were compared within EAs and PAs, and EAs were compared with PAs in both phases. We analyzed two post‐arousal windows: (a) *t* = 0–48 s (full period) and (b) *t* = 25–48 s (later half), to cover overall and late HR changes. Unimodal distribution of median HR across patients and timepoints was assessed using Hartigan's dip test for EAs and PAs. Variability of HR dynamics was quantified as IQR of HR across patients and timepoints, and compared between EAs and PAs using permutation tests and ROC analysis for both time windows. Depending on data distribution, continuous variables are reported as mean ± SD or median (IQR) with ranges given in square brackets. Data processing and statistical analyses were performed using MATLAB (version 9.11, The MathWorks Inc., 2021) and R (version 4.3.1, R Core Team, 2023).

## 3 RESULTS

### 3.1 Dataset

EAs were reported in 207 of 3087 patients undergoing video‐EEG monitoring at the Epilepsy Center Freiburg (January 2010–January 2023), corresponding to a prevalence of 6.7%. Twenty‐four patients had simultaneous iEEG and scalp EEG. Four patients were excluded due to comorbidity (*n* = 1) or misclassification of seizures as EAs (*n* = 3).

The present study included 20 patients (12 female, 8 male; mean age 29.3 ± 13.3 [12–57] years), monitored over 159 nights with 507 recorded, annotated, and analyzed epileptic seizures, including 133 EAs (median 2.5 (IQR 6.8) [1–49] per patient), representing 26.2% of all recorded seizures (median 16.7% (IQR 47.4) [2.2–90.9] per patient). Five patients exhibited a high burden of EAs (≥10 EAs). Twelve patients had temporal lobe epilepsy (TLE), four frontal lobe epilepsy (FLE), and four multifocal epilepsy (Table 1). After presurgical diagnostics, 14 patients (70%) underwent resective epilepsy surgery.

**TABLE 1:** Population.

Patient	Sex	Age (years)	Epilepsy	Side	Etiology	EA (*n*)
1	f	18	FLE	r	FCD^a^	4
2	m	37	MF	bi	FCD^b^, schizencephaly^b^, PVH^b^	49
4	f	22	FLE	l	FCD^b^	11
5	m	21	TLE	bi	Encephalocele^b^	2
6	f	51	TLE	r	Non‐lesional^b^	1
8	m	20	TLE	r	HS^a^	1
9	f	48	TLE	r	Meningoencephalocele^a^	1
10	m	29	FLE	r	mMCD^a^	2
11	f	15	MF	bi	FCD^a^	4
12	m	33	TLE	r	FCD^a^, HS^a^	4
14	f	12	TLE	r	Splenial and septal malformation^b^, malrotation of hippocampi^b^	1
15	m	12	TLE	r	Unknown^b^	2
16	f	33	MF	bi	Ganglioglioma^b^	1
17	f	22	FLE	r	FCD^b^	11
18	f	57	TLE	bi	Non‐lesional^a^	1
19	f	40	TLE	bi	HS^a^	1
20	m	43	TLE	bi	Non‐lesional^b^	10
22	m	16	MF	l	Unknown^b^	7
23	f	29	TLE	bi	mMCD^a^	3
24	f	28	TLE	bi	Non‐lesional^b^	17

Abbreviations: bi, bi‐hemispheric; EA, epileptic arousals included; f, female; FCD, focal cortical dysplasia; FLE, frontal lobe epilepsy; HS, hippocampal sclerosis; l, left; m, male; MF, multifocal; mMCD, mild malformation of cortical development; PVH, periventricular heterotopy; r, right; TLE, temporal lobe epilepsy.

^a^ Etiology determined by pathology.

^b^ Etiology determined by MR imaging. Patient numbering reflects the original cohort; excluded patients (3, 7, 13, and 21) are omitted.

### 3.2 iEEG coverage

A total of 188 intracranial depth electrodes were stereotactically implanted (127 right, 61 left, mean 9.4 ± 2.5 [2–13] per patient), each with 4–14 contacts, totaling 1474 contacts (Figure 3A), that covered 49 anatomic regions, irrespective of hemisphere. Each anatomic region was sampled by a median of 13 contacts (IQR 30 [1–252]), across a median of 4 patients (IQR 5 [1–16]).

**FIGURE 3:**
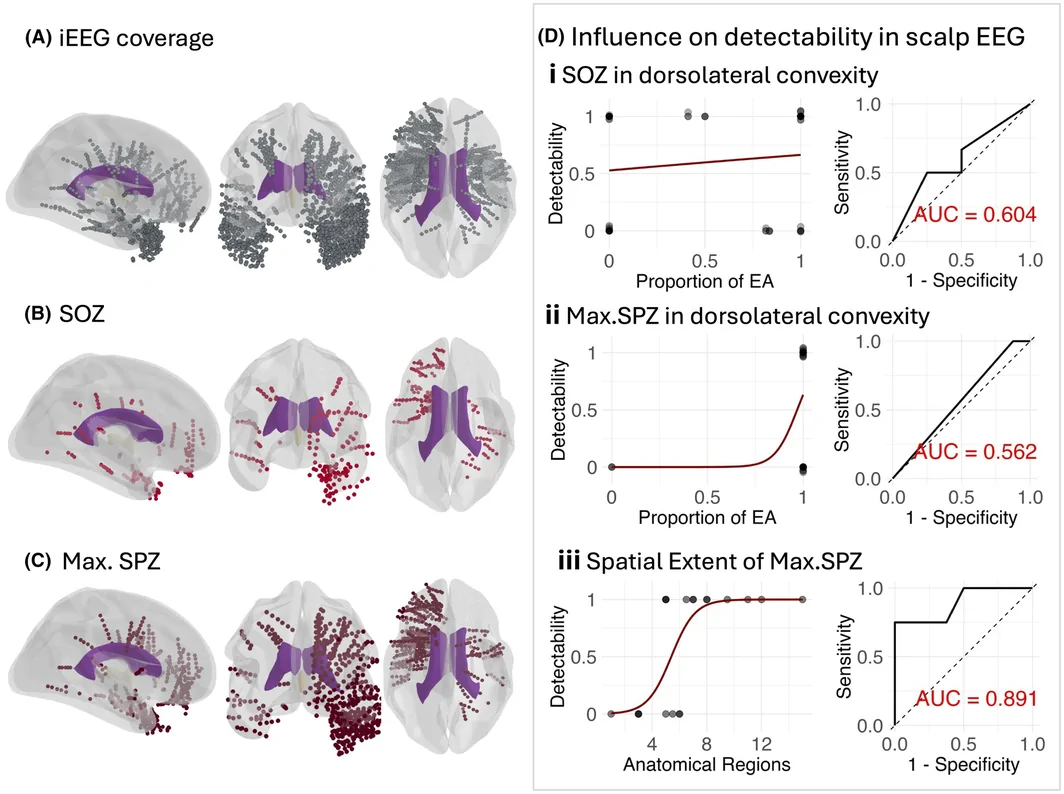
Relationship between iEEG ictal activity and scalp EEG detectability. (A) All included intracranially implanted electrodes (*n* = 188) with MNI coordinates of each electrode contact (*n* = 1474) from which iEEG was recorded, projected on a glass brain in sagittal, frontal, and transverse views. Lateral ventricles in purple for spatial orientation. (B) Intracranial electrode contacts involved in SOZ and (C) max SPZ, each in sagittal, frontal, and transverse views. (D) Influence on the detectability of ictal activity in scalp EEG. Logistic regression models (left panels) predict scalp EEG detectability (detectable vs. not detectable) from three predictors: (i) Proportion of EAs per patient with SOZs in dorsolateral convexity, (ii) proportion of EAs per patient with max SPZ in dorsolateral convexity, and (iii) median number of anatomically distinct regions per patient involved in max SPZ. Model performances were evaluated using ROC curves (right panels). Detectability of EAs in scalp EEG does not correlate with the localization of SOZ or max SPZ in dorsolateral convexity but is influenced by the spatial extent of max SPZ. The ROC‐AUC of 0.891 confirms the high discriminative ability of the model. AUC, area under the curve; EA, epileptic arousal; EEG, electroencephalography; iEEG, intracranial EEG; max SPZ, maximum seizure propagation zone; MNI, Montreal Neurological Institute; ROC, receiver operating characteristic; SOZ, seizure‐onset zone.

### 3.3 Seizure onset zone

At a patient‐level across all seizure types, SOZ was identifiable in all patients (*n* = 20). In five patients (25%), additional SOZ outside the implanted region was suspected due to incomplete sampling of habitual seizure types (*n* = 3) or incomplete spatial coverage of iEEG (n = 3); in one patient both applied.

Patient‐level characteristics of EAs, including SOZ, max SPZ, scalp EEG detectability, comparison to other seizure types, and surgical outcome, are summarized in Table 2.

**TABLE 2:** Seizure‐onset zone in epileptic arousals and other seizure types in iEEG, maximum seizure propagation zone on iEEG and detectability of ictal activity on scalp EEG, and surgery outcome.

Case	SOZ in iEEG				Jaccard similarity	Max SPZ in iEEG			Detectability of ictal activity in scalp EEG	Surgery outcome
	EA			Other seizure types		EA				
	Depth electrode contacts (*n*)	Number of anatomic regions (*n*)	Anatomic regions	Anatomic regions		Depth electrode contacts (*n*)	Number of anatomic regions (*n*)	Anatomic regions		*Engel*
1	14	5	FP, FMC, FOpC, IFG, MFG	IFG, MFG	0,4	47	13	FP, PCG, FOpC, IFG, MFG, CC, CR, FOrC, JPLC, PreCG, SFG, CG, IC	✓	Ia
2	19	4	PostCG, CR, SLF, PreCG	–^a^	–	44	5	CR, SLF, PreCG, MFG, PostCG	✕	–
4	3	2	PreCG, SFG	SFG, PreCG	1	4	2	SFG, PreCG	✕	–
5	8	4	TP, EC, TFC, H	TP, EC, TFC, A, H^a^, ^b^	0,8	34	9	TP, ITG, MTG, TFC, A, PP, STG, EC	✓	Ia
6	1	1	EC	EC, TP, MTG, COC, PreCG^b^	0,2	13	6	TFC, TP, A, PP, H, PHG	✕	–
8	4	3	EC, A, H	EC, H, A, PHG	0,75	9	5	TFC, PHG, IC, FOrC, IFG	✓	Ia
9	2	1	H	L, TP	0	21	8	L, TP, TFC, ITG, MTG, A, PHG, TOFC	✓	Ia
10	3	2	FOrC, FP	FOrC, FP	1	58	12	FMC, CR, FP, MFG, FOrC, SCC, PCG, CC, CG, JPLC, SFG	✓	Ia
11	8	3	LOC, CCx, OP	LOC, CCx, PostCG^a^	0,5	39	12	PCC, LOC, CCx, OP, LG, PostCG, JPLC, PreCG, SFG, SPL, SMG, AG	✓	IId
12	13	5	TP, H, PHG, STG, MTG	STG	0,2	56	14	TP, A, poMTG, MTG, TFC, ITG, IC, PP, H, PHG, STG, AG, LOC	✓✕	Ia
14	3	1	H	H	1	9	5	H, A, TFC, TP, MTG	✕	IIb
#15	2	1	TP	H, TFC, TP^b^	0,33	64	15	FMC, PCG, FP, SCC, FOrC, MFG, TP, A, TFC, ITG, MTG, H, STG, AG	✓	Ic
16	2	1	H	FP, H, A, EC	0,2	11	5	H, TFC, ITG, EC, A	✓	IVb
17	37	7	IC, FOpC, IFG, CG, FP, PCG, FMC	PHG, A, TFC, FP	0,1	67	10	IFG, FP, CG, PCG, SFG, FMC, IC, FOpC, MFG	✕	IVa
18	4	4	EC, TFC, A, PHG	A, H, EC, PHG, TFC, TP	0,57	39	7	TP, TFC, ITG, MTG, PHG, H, EC	✓	Ia
19	2	2	A, H	A, H, FFG, TP	0,4	3	3	EC, H, FFG	✕	Ia
20	4	2	H, PHG	TP, H	0,2	23	11	H, PT, TP, MTG, TFC, PHG, PP, A, STG, ITG	✕	–
22	26	7	MOG, LOC, TFC, ITG, MTG, SPL, AG	SPL, MOG, LOC	0,43	37	13	TOFC, LOC, H, PHG, TFC, ITG, MTG, PostCG, PreCG, PCC, COC, STG, AG	✓	–
23	7	4	TP, MTG, EC, PHG	EC, PHG, TP, TFC, A, H	0,43	12	6	TP, EC, PHG, TFC, MTG, H	✓	IIb
24	11	7	H, A, PHG, TFC, TP, ITG, SS	H, PHG, A, ITG	0,38	38	11	PHG, TP, EC, TFC, ITG, MTG, H, SS, A, STG	✓	–

*Note*: Anatomic regions (sorted alphabetically): A, amygdala; AG, angular gyrus; CC, corpus callosum; CCx, cuneal cortex; CG, cingulate gyrus; COC, central opercular cortex; CR, corona radiata; EC, entorhinal cortex; FFG, fusiform gyrus; FMC, frontal medial cortex; FOpC, frontal operculum cortex; FOrC, frontal orbital cortex; FP, frontal pole; H, hippocampus; IC, insular cortex; IFG, inferior frontal gyrus; ITG, inferior temporal gyrus; JPLC, juxtapositional lobule cortex; L, lesion; LG, lingual gyrus; LOC, lateral occipital cortex; MFG, middle frontal gyrus; MOG, middle occipital gyrus; MTG, middle temporal gyrus; OP, occipital pole; PCC, precuneus cortex; PCG, paracingulate gyrus; PHG, parahippocampal gyrus; poMTG, pole of middle temporal gyrus; PostCG, postcentral gyrus; PP, planum polare; PreCG, precentral gyrus; PT, planum temporale; SCC, subcallosal cortex; SFG, superior frontal gyrus; SLF, superior longitudinal fasciculus; SMG, supramarginal gyrus; SPL, superior parietal lobule; SS, sagittal stratum; STG, superior temporal gyrus; TFC, temporal fusiform cortex; TOFC, temporal occipital fusiform cortex; TP, temporal pole.

Abbreviations: EA, epileptic arousal; EEG, electroencephalography; iEEG, intracranial EEG; Max SPZ, maximum seizure‐propagation zone; SOZ, seizure‐onset zone.

^a^ Indication for additional SOZ outside of sampled region due to incomplete sampling of habitual seizure types or.

^b^ Due to incomplete sampling of anatomical regions. Jaccard similarity = |A ∩ B|/|A ∪ B|, where A and B are sets of anatomic regions. ✓: detectable, ✕: not detectable.

Of 1474 total electrode contacts, 173 (11.7%) exhibited ictal activity at seizure onset of EAs, identifying 30 anatomic regions as SOZ of EAs (Table 3 Figure 3A).

**TABLE 3:** Spatial characteristics and scalp EEG detectability of EA.

A. SOZ and max SPZ of EA				
Parameter		SOZ	Max SPZ	
Total	Electrode contacts *n* (%)	173 (11.7%)	628 (42.6%)	
	Anatomical regions *n*	30	43	
Per patient	Electrode contacts^ª^	4 (8.75) [1–37]	31 ± 20.7 [3–67]	
	Anatomic regions^ª^	3 (2.5) [1–7]	8.6 ± 3.9 [2–15]	
Per EA	Electrode contacts^ª^	3 (3) [1–29]	13 (18) [1–64]	
	Anatomic regions^ª^	2 (2) [1–7]	5 (5) [1–15]	
B. Detectability of ictal activity in scalp EEG				
**Parameter**		**Detectable**	**Not detectable**	**Both**
Per EA	*n* (%)	44 (33.1%)	89 (66.9%)	–
Per patient	*n* (%)	11 (55.0%)	8 (40.0%)	1 (5%)
**Consistency**		**Consistent**		**Not consistent**
Per patient	*n* (%)	19 (95%)		1 (5%)
C. Predictors of scalp EEG detectability (logistic regression)				
**Predictor**		**OR [95% CI]**	***p***	**ROC‐AUC [95% CI]**
Dorsolateral convexity involvement	SOZ	1.77 [0.25–13.65]	0.571	0.604 [0.357–0.851]
	Max. SPZ	6.00 [0.51–145.52]	0.183	0.562 [0.44–0.685]
Spatial extent (anatomic regions)	Max. SPZ	3.16 [1.42–16.01]	0.046	0.891 [0.75–1]

Abbreviations: AUC, area under the curve; CI, confidence interval; EA, epileptic arousal; EEG, electroencephalography; IQR, interquartile range; Max SPZ, maximum seizure‐propagation zone; OR, odds ratio; ROC, receiver operating characteristic; SD, standard deviation; SOZ, seizure‐onset zone.

^ª^ Depending on data distribution, statistics are reported as mean ± SD [range] or median (IQR) [range].

Mesial temporal structures were most frequently involved in SOZs of EAs, including hippocampus, parahippocampal gyrus, amygdala, and entorhinal cortex. The temporal pole and fusiform cortex were also commonly implicated. Extratemporal SOZs were less frequent involving the precentral gyrus and frontal pole. Occasionally, SOZs occurred in cingulate, insular, and occipital regions (Figure S2).

In one patient, no seizure types other than EAs were recorded, precluding SOZ comparison with other seizure types. Therefore, the following analysis includes 19 patients (95%). Comparison of SOZs between EAs and other seizure types revealed a mean Jaccard similarity index of 0.47 (± 0.31 [0–1]). Most patients (*n* = 15, 78.9%) exhibited partial overlap between the SOZ of EA and other seizure types, three (15.8%) identical, and one (5.3%) no overlap.

### 3.4 Maximum seizure‐propagation zone

Maximal seizure propagation of EAs was observed in 628 electrode contacts (42.6%), covering 43 anatomic regions as max SPZ (Table 3A, Figure 3C). Mesial temporal structures were frequently recruited, including hippocampus, parahippocampal gyrus, amygdala, and entorhinal cortex. Lateral temporal regions were also commonly involved, particularly the temporal fusiform cortex; middle, and inferior temporal gyri; and the temporal pole. Propagation to frontal regions occurred less often and involved mainly the precentral gyrus, frontal pole, and middle frontal gyrus. Parietal, insular, midline, and occipital regions were rarely involved (Figure S3). The median duration of ictal activity in iEEG during EAs was 13 s (IQR 39 [4–174]).

### 3.5 Detectability of epileptic arousals

Of the 133 included EAs, 89 (66.9%) from 9 patients showed no detectable ictal activity in scalp EEG, whereas 44 EAs (33.1%) from 12 patients were detectable; one patient contributed to both groups. Detectability was consistent in 19 patients (95%), meaning that within each patient, either all EAs were detectable or none were detectable on scalp EEG (Table 3B).

### 3.6 Influence of dorsolateral convexity involvement on ictal detectability in scalp EEG

Across all EAs, the dorsolateral convexity was involved in the SOZs of 95 EAs (71.4%) from 12 patients, and in the max SPZ of 132 EAs (99.2%) from 19 patients. Logistic regression showed that dorsolateral convexity involvement in SOZ and max SPZ was not associated with detectability of ictal activity in scalp EEG (Table 3, Figure 3Di,ii).

### 3.7 Influence of spatial extent of seizure propagation on ictal detectability in scalp EEG

EAs detectable by scalp EEG showed broader max SPZ than non‐detectable EAs. They involved more iEEG electrode contacts (median 23.5 (IQR 17.2) [9–64] vs 10 (IQR 8) [1–48]) and more anatomic regions (median 9 (IQR 4) [4–15] vs 3 (IQR 2) [1–9]). Scalp EEG detectability of ictal activity was significantly associated with the median number of anatomic regions with ictal activity in iEEG at max SPZ. Increasing the involvement of anatomic regions corresponded to higher odds of detection (Table 3C, Figure 3Diii).

### 3.8 Sleep stage distribution and arousal types of epileptic arousals

EAs occurred predominantly during N2 sleep (116 (87%) in 19 patients) and less frequently in N3 sleep (17 (13%) in 6 patients); none were recorded during REM sleep. Most EAs were fast arousals (79 (59%) in 16 patients), followed by intermediate (37 (28%) in 8 patients) and slow arousals (10 EAs (7.5%) in 3 patients). In one patient, seven EAs were unclassifiable due to muscle artifacts. Among the remaining 19 patients, 12 (63.2%) consistently showed a single arousal type, whereas 7 (36.8%) showed multiple types.

### 3.9 Influence of epileptic arousals and physiological arousals on heart rate

HR analysis included 79 EAs and 76 PAs during N2 from 15 patients; 5 patients were excluded due to poor ECG signal quality (*n* = 3), clinical testing during or after the seizure (*n* = 1), or EAs outside N2 sleep (*n* = 1). Pre‐arousal HRs did not differ between EAs and PAs. HRs increased significantly after the arousal onset in scalp EEG in EAs and PAs (Wilcoxon rank‐sum test, *p* < 0.001 for both), but showed a higher median increase and greater variability in EAs (*p* < 0.001). In the time window *t* = 0–48 s after arousal onset, median HR per patient showed unimodal distribution in EAs and PAs. However, in the time window *t* = 25–48 s after arousal onset, EAs exhibited significant multimodal distribution and greater variability, quantified as IQR of median HR across patients at each timepoint (Hartigan's dip test, *p* < 0.001, permutation test, *p* = 0.007), enabling highly accurate discrimination from PA (ROC‐AUC = 0.995) (Figure 4, Table S1).

**FIGURE 4:**
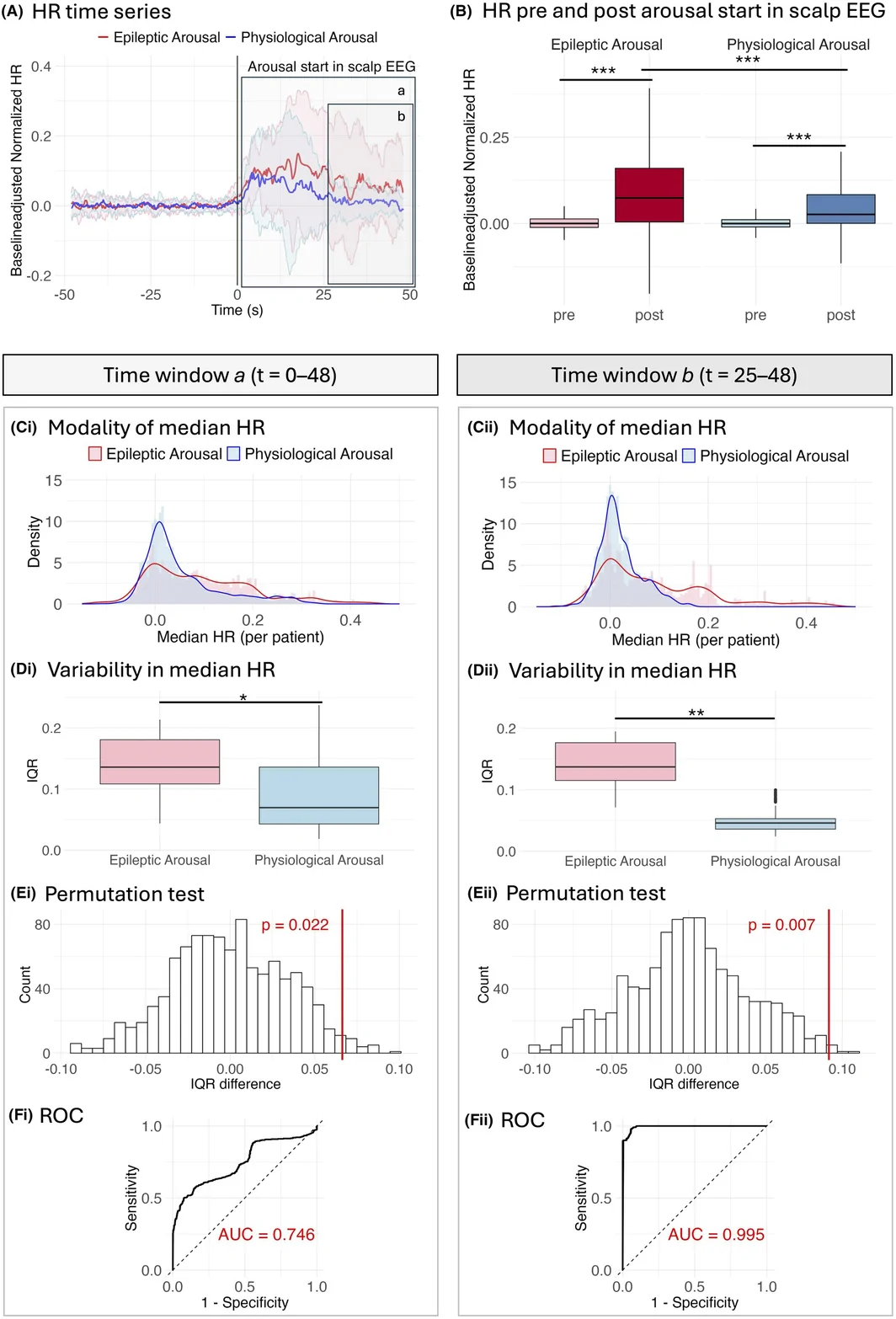
Modulation of HR in EAs and PAs. HR is baseline‐adjusted and normalized with medians calculated per patient and across patients. (A) HR time series (median ± IQR) aligned to arousal onset at t = 0, as seen on scalp EEG. (B) Compared to baseline (median HR 48 s pre‐arousal), both EAs and PAs show significant increase in HR (Wilcoxon rank‐sum test, *p* < 0.001). Pre‐arousal HR does not differ between EA and PA (Wilcoxon rank‐sum test, *p* = 0.958). However, HR post‐arousal is significantly higher in EAs with greater variability compared to PAs (Wilcoxon rank‐sum test, *p* < 0.001). Two time windows are analyzed: (A) 0–48 s and (B) 25–48 s. (C) The distribution of median HR does not deviate significantly from unimodality in both EA (Hartigan's dip test, *p* = 0.29) and PA (Hartigan's dip test, *p* = 0.91) during time window A (0–48 s; Ci). However, in time window B (25–48 s; Cii), EA distribution is no longer unimodal (Hartigan's dip test, *p* < 0.001), in contrast to PA (Hartigan's dip test, *p* = 0.77). (D–F) Variability in HR is measured as IQR of median HRs across patients at every timepoint. EAs show significantly higher variability in HR compared to PAs. This effect is even more pronounced in time window B with permutation test *p* = 0.007 with ROC‐AUC of 0.995 (Dii, Eii, Fii) compared to time window A with *p* = 0.022 with ROC‐AUC of 0.746 (Di, Ei, Fi). Statistical significance is indicated as follows: *p < 0.05, **p < 0.01, ***p < 0.001. AUC, area under the curve; EA, epileptic arousal; EEG, electroencephalography; HR, heart rate; IQR, interquartile range; PA, physiological arousal; ROC, receiver operating characteristic.

## 4 DISCUSSION

### 4.1 Key findings

EAs are common yet underrecognized. In this study, we systematically characterized EAs. Among patients with EAs, they account for over 25% of all recorded seizures. EAs arise from partially distinct epileptic networks and exhibit a distinct autonomic signature, supporting their classification as separate entity.

Our findings reveal a diagnostic gap: Using simultaneous iEEG and scalp EEG, two‐thirds of EAs showed no detectable ictal activity in scalp EEG and thus might be misclassified as PAs when relying on scalp EEG alone. In 95% of patients, EAs were either all detectable or undetectable. Detectability increased with broader SPZs in iEEG.

### 4.2 Epileptic arousals as distinct seizure type

Although various sleep‐related epileptic seizures are accompanied by arousals,^26^ EAs represent a distinct seizure type in which arousal is the sole semiological manifestation. Their SOZ shows only partial spatial overlap with other seizure types within the same patient, suggesting partially distinct epileptic networks involved in their generation. Moreover, EAs exhibit a specific autonomic signature.

This distinction suggests that differentiation of EAs from other epileptic and non‐epileptic events may be clinically relevant for a diagnosis of epilepsy, in the context of presurgical evaluation and epilepsy surgery planning, and for treatment outcome assessment. Apart from seizures accompanied by arousals and PAs, key differential diagnoses include subclinical electrographic events, autonomic seizures (e.g., isolated ictal tachycardia), paroxysmal arousals and minor motor events in sleep‐related hypermotor epilepsy (SHE), and confusional arousals as disorder of arousal (DOA).

### 4.3 Conceptual framework: How epileptic networks induce arousal

Although the precise pathophysiology of EAs remains unclear, we propose that ictal activity induces arousals via “top‐down” cortical pathways. Previous studies have demonstrated that ictal activity can trigger arousals during sleep.^7^, ^9^ This aligns with emerging models of sleep regulation in which cortical regions influence arousal via cortico‐thalamic pathways,^27^ complementing the classical “bottom‐up” activation by the ascending reticular activating system (ARAS).^28^ In EAs, arousal represents the primary semiology, suggesting direct modulation of arousal‐promoting systems. By contrast, in seizures with complex semiology, arousals may occur secondarily to sensory, motor, or autonomic manifestations.^7^ Arousals frequently represent the earliest observable sign of nocturnal seizures, suggesting early ARAS involvement.^8^ Single‐case studies about seizures with arousing effects identified SOZs in regions such as the mesial orbitofrontal cortex^13^ and anterior cingulate gyrus.^14^ In contrast, we identified SOZs and SPZs across numerous anatomic regions, suggesting structural or functional connectivity with arousal‐ or sleep‐regulatory networks as symptomatogenic zone of EAs. This may involve excitation of arousal‐promoting systems or inhibition of sleep‐promoting systems. These findings support the concept of a broader, shared network architecture linking sleep, arousal, and epileptic activity,^29^ in which diverse cortical and subcortical regions interact with arousal‐regulating neuronal populations to mediate transitions from sleep toward wakefulness.^30^ Redundancy of arousal‐promoting systems^31^ and increased excitatory neurotransmission through ictal activity^32^ may enable heterogenous brain regions to induce arousals in EAs, functionally linking cortical hyperexcitability with hyperarousability. Moreover, this model requires moving beyond linear, unidirectional causality: although ictal activity can elicit arousals, arousal states may in turn modulate ictal activity. Evidence from slow oscillations in sleep^33^ and CAP as a marker of sleep instability^34^ link fluctuating arousal levels to the occurrence of interictal discharges and seizures.

### 4.4 Autonomic signature of epileptic arousals: Why heart rate dynamics are more variable

Although both EAs and PAs modulate HR, EAs exhibit greater variability and multimodal distribution, consistent with earlier observations that HR responses differ between seizures accompanied by arousals and PAs.^14^, ^17^ This pattern likely reflects the combined effects of heterogeneity in both arousals^1^ and seizures.^35^ Recent iEEG studies demonstrate spatial variability in arousal expression across brain regions,^36^ with autonomic responses scaling with EEG desynchronization across arousal types,^37^ arousal intensity, and components, such as motor activation.^38^ In our cohort, SOZs and SPZs were distributed across multiple anatomic regions, which may contribute to variability in HR responses, consistent with prior findings that autonomic changes depend on seizure localization and propagation in ictal tachycardia.^39^ The convergence of these processes in EAs likely amplifies fluctuations in autonomic output, resulting in the observed variability and multimodal distribution.

### 4.5 A diagnostic gap and its clinical implications

The reliable detection of EAs and precise distinction from PAs require invasive diagnostics such as iEEG, which is rarely performed for symptoms of unclear sleep fragmentation or daytime sleepiness. Therefore, EAs may remain undetected in many patients. Detectability of EAs on scalp EEG in our cohort does not significantly correlate with localization in the dorsolateral convexity, but with the spatial extent of the max SPZ. This observation is consistent with established biophysical principles of EEG signal generation, including neural synchrony and the orientation of cortical dipoles.^40^ Within patients, EAs mostly were either consistently detectable or undetectable by scalp EEG. Thus, in a non‐invasive setting relying solely on scalp EEG, clinicians are not merely missing single seizures but potentially fail to identify entire patients whose EAs remain invisible to scalp EEG.

Our cohort consisted of patients with drug‐resistant focal epilepsy, all of whom exhibited other seizure types in addition to EA. It remains unknown whether EAs also occur in generalized epilepsy. EAs as sole manifestations of epilepsy may represent an underrecognized patient group whose symptoms may remain unexplained or misattributed.

Undetected EAs may contribute to the vicious cycle perpetuating sleep fragmentation and epileptic activity,^11^ worsening seizure control,^41^ and increasing risk for sudden unexpected death in epilepsy (SUDEP),^42^ neuropsychiatric comorbidities,^43^ cognitive impairment,^44^ and reduced quality of life.^45^, ^46^ This underscores the importance of recognizing and accurately diagnosing EAs.

### 4.6 Study limitations

The study was conducted under standard presurgical epilepsy‐monitoring protocols. Determination of the SOZ could be limited by incomplete sampling of seizure types and spatial coverage. Frequency and occurrence of EAs may depend on the reduction of antiseizure medication performed during monitoring. No full polysomnography was recorded, and sleep conditions were not standardized: Multiple clinical factors, including sleep deprivation, titration of antiseizure medication, recent implantation of intracranial depth electrodes, intercurrent seizures, and environmental disturbances, may have influenced both EAs and PAs. Interictal epileptic activity during PAs could not be excluded.

The analysis relied on retrospective analysis of clinically annotated seizures. Due to standard clinical workflows, full screening for all EAs was not feasible. EA determination based on inter‐rater concordance may underestimate the occurrence of very subtle EAs. Potential selection bias may have occurred during PA screening, favoring arousals with more pronounced cortical, motor, and autonomic components. Although HR dynamics differed at the group level, the available number of events per patient was insufficient to evaluate patient‐specific classification performance. Despite a heterogeneous, relatively small and unbalanced dataset, our study represents the largest and most systematically characterized cohort of EAs to date.

## 5 CONCLUSION

Our findings support the recognition of EAs as a distinct seizure type and their integration into future ILAE classifications to raise clinical and scientific awareness.

We provide insights into neural and autonomic mechanisms of EAs. Future work should explore the impact of EAs on sleep architecture, seizure control, and daytime functioning, and compare EAs to other seizure types regarding network involvement and HR dynamics. These distinctive features may provide a first step toward overcoming the diagnostic challenges of this neglected clinical entity.

## AUTHOR CONTRIBUTIONS

L.F.: Conceptualization, methodology, investigation, data curation, software, formal analysis, writing – original draft. N.P.: Conceptualization, investigation, writing – review and editing. S.K.: Validation, writing – review and editing. N.Z.: Validation, writing – review and editing. A.B.: Data curation, writing – review and editing. P.C.R.: Implantation of intracranial electrodes, writing – review and editing. D.R.: Conceptualization, resources, supervision, writing – review and editing. M.K.: Conceptualization, methodology, validation, supervision, writing – review and editing. A.S.B.: Conceptualization, resources, validation, supervision, writing – review and editing. All authors have approved the final article.

## CONFLICT OF INTEREST STATEMENT

L.F. has received travel grants from the German Society of Neurology (DGN) and the Prof. Dr. Peter and Jytte Wolf Foundation for Epilepsy, and a poster award from the German Society for Clinical Neurophysiology (DGKN). N.P. has received EJPRD Research Mobility Fellowship and ERN Research Mobility Fellowship from the European Joint Programme Rare Diseases (EJPRD). P.C.R. has received research support from the Else Kröner Fresenius Foundation, Fraunhofer Foundation (ATTRACT), German Ministry for Economic Affairs and Energy, and Medical Faculty of the University of Freiburg. He has received personal honoraria for lectures or advice from Boston Scientific, Brainlab, Inomed, and Fraunhofer Foundation, and is a consultant to Boston Scientific, Brainlab, and Inomed. A.S.B. has received honoraria for lectures or advice from Angelini Pharma, EISAI, JAZZ Pharma, Precisis, UCB, and UNEEG, unrelated to the topic of the manuscript. The remaining authors have no conflicts of interest to disclose.

## ETHICS STATEMENT

We confirm that we have read the Journal's position on issues involved in ethical publication and affirm that this report is consistent with those guidelines.

## Supporting information


**Figure S1:** Physiological arousal in simultaneous intracranial and scalp EEG. A fast‐type arousal during N2 sleep becomes apparent on scalp EEG (vertical line). No ictal activity is detectable in either intracranial or scalp EEG. EEG, electroencephalography; iEEG, intracranial EEG; ECG, electrocardiography.
**Figure S2.** Seizure‐onset zones in epileptic arousals. Visually identified ictal activity at seizure onset in iEEG channels mapped to associated anatomic localization. For each anatomic localization, the numbers of depth electrode contacts (thick pink bar), seizures (thin dark red bar), and patients (red points) were counted. Thirty anatomic regions were identified as part of the seizure‐onset zone. iEEG, intracranial electroencephalography.
**Figure S3.** Maximum seizure‐propagation zones in epileptic arousals. Visually identified ictal activity at maximum extent of seizure propagation in iEEG channels mapped to associated anatomic localization. For each anatomic localization, the numbers of depth electrode contacts (think dark red bar), seizures (thin black bar), and patients (red points) were counted. iEEG, intracranial electroencephalography. 0100200300 0 5 10 4 5.
**Table S1.** Influence of epileptic arousals and physiological arousals on heart rate.

## Data Availability

Anonymized data that support the findings of this study are available from the authors upon reasonable request.

## References

[1] Halász P , Terzano M , Parrino L , Bódizs R . The nature of arousal in sleep. J Sleep Res. 2004;13:1–23. 10.1111/j.1365-2869.2004.00388.x 14996030

[2] Riemann D , Dressle RJ , Benz F , Spiegelhalder K , Johann AF , Nissen C , et al. Chronic insomnia, REM sleep instability and emotional dysregulation: a pathway to anxiety and depression? J Sleep Res. 2025;34:e14252. 10.1111/jsr.14252 PMC11911052 38811745

[3] Halász P , Szűcs A , Mutti C , Parrino L . Disorders of arousal and sleep‐related hypermotor epilepsy — overview and challenges night is a battlefield of sleep and arousal promoting forces. Neurol Sci. 2022;43:927–937. 10.1007/s10072-021-05857-0 34984571

[4] Fisher RS , Boas WVE , Blume W , Elger C , Genton P , Lee P , et al. Epileptic seizures and epilepsy: definitions proposed by the international league against epilepsy (ILAE) and the International Bureau for Epilepsy (IBE). Epilepsia. 2005;46:470–472. 10.1111/j.0013-9580.2005.66104.x 15816939

[5] Lüders HO , Najm I , Nair D , Widdess‐Walsh P , Bingman W . The epileptogenic zone: general principles. Epileptic Disord. 2006;8(Suppl 2):S1–S9. 10.1684/j.1950-6945.2006.tb00204.x 17012067

[6] Frauscher B , Gotman J . Sleep, oscillations, interictal discharges, and seizures in human focal epilepsy. Neurobiol Dis. 2019;127:545–553. 10.1016/j.nbd.2019.04.007 30981828

[7] Peter‐Derex L , Klimes P , Latreille V , Bouhadoun S , Dubeau F , Frauscher B . Sleep disruption in epilepsy: ictal and interictal epileptic activity matter. Ann Neurol. 2020;88:907–920. 10.1002/ana.25884 32833279

[8] Malow B , Varma N . Seizures and arousals from sleep‐which comes first? Sleep. 1995;18:783–786. 10.1093/sleep/18.9.783 8638072

[9] Malow A , Bowes RJ , Ross D . Relationship of temporal lobe seizures to sleep and arousal: a combined scalp‐intracranial electrode study. Sleep. 2000;23:1–4. 10.1093/sleep/23.2.1j 10737340

[10] Beniczky S , Trinka E , Wirrell E , Abdulla F , Al Baradie R , Alonso Vanegas M , et al. Updated classification of epileptic seizures: position paper of the International League Against Epilepsy. Epilepsia. 2025;66:1804–1823. 10.1111/epi.18338 PMC12169392 40264351

[11] Sheybani L , Frauscher B , Bernard C , Walker MC . Mechanistic insights into the interaction between epilepsy and sleep. Nat Rev Neurol. 2025;21:177–192. 10.1038/s41582-025-01064-z 40065066

[12] Awad AM , Lüders HO . Hypnopompic seizures. Epileptic Disord. 2011;12:270–274. 10.1684/epd.2010.0336 21030341

[13] Alisali M , Joshi S , Ganne C , Vashin V , Pati S . Localization of hypnopompic seizures – a stereo EEG study. Epilepsy Behav Rep. 2024;28:100729. 10.1016/j.ebr.2024.100729 PMC11621591 39651453

[14] Toth E , Chaitanya G , Pogwizd M , Pizarro D , Ilyas A , Pogwizd S , et al. Dynamics of seizure‐induced behavioral and autonomic arousal. Clin Auton Res. 2020;29:205–209. 10.1007/s10286-018-0543-x 30008021

[15] Nobili L , Frauscher B , Eriksson S , Gibbs SA , Halasz P , Lambert I , et al. Sleep and epilepsy: a snapshot of knowledge and future research lines. J Sleep Res. 2022;31:e13622. 10.1111/jsr.13622 PMC9540671 35487880

[16] Castillo Rodriguez M d LA , Brandt A , Schulze‐Bonhage A . Differentiation of subclinical and clinical electrographic events in long‐term electroencephalographic recordings. Epilepsia. 2022;00:1–12. 10.1111/epi.17401 36008142

[17] Kubota T , Thyagaraj S , Gia Huynh H , Kanubhai Gajera P , Awori V , Zande JL , et al. Distinction between epileptic and non‐epileptic arousal by heart rate change. Epilepsy Behav. 2023;148:109487. 10.1016/j.yebeh.2023.109487 37897862

[18] Zijlmans M , Zweiphenning W , van Klink N . Changing concepts in presurgical assessment for epilepsy surgery. Nat Rev Neurol. 2019;15:594–606. 10.1038/s41582-019-0224-y 31341275

[19] Evans AC , Kamber M , Collins DL , MacDonald D . An MRI‐based probabilistic atlas of neuroanatomy. In: Shorvon SD , Fish DR , Andermann F , Bydder GM , Stefan H , editors. Magnetic resonance scanning and epilepsy. Boston, MA: Springer US; 1994. p. 263–274. 10.1007/978-1-4615-2546-2_48

[20] Desikan RS , Ségonne F , Fischl B , Quinn BT , Dickerson BC , Blacker D , et al. An automated labeling system for subdividing the human cerebral cortex on MRI scans into gyral based regions of interest. NeuroImage. 2006;31:968–980. 10.1016/j.neuroimage.2006.01.021 16530430

[21] Faria AV , Joel SE , Zhang Y , Oishi K , Van Zjil PCM , Miller MI , et al. Atlas‐based analysis of resting‐state functional connectivity: evaluation for reproducibility and multi‐modal anatomy–function correlation studies. NeuroImage. 2012;61:613–621. 10.1016/j.neuroimage.2012.03.078 PMC3358461 22498656

[22] Wakana S , Caprihan A , Panzenboeck MM , Fallon JH , Perry M , Gollub RL , et al. Reproducibility of quantitative tractography methods applied to cerebral white matter. NeuroImage. 2007;36:630–644. 10.1016/j.neuroimage.2007.02.049 PMC2350213 17481925

[23] Troester MM , Quan SF , Berry RB , Plante DT , Abreu AR , Alzoubaidi M , et al. The AASM manual for the scoring of sleep and associated events: rules, terminology and technical specifications. Version 3 ed. Darien, Illinois: American Academy of Sleep Medicine; 2023.

[24] Terzano MG , Parrino L , Smerieri A , Chervin R , Chokroverty S , Guilleminault C , et al. Atlas, rules, and recording techniques for the scoring of cyclic alternating pattern (CAP) in human sleep. Sleep Med. 2002;3:187–199. 10.1016/s1389-9457(02)00003-5 14592244

[25] Sedghamiz H . Matlab Implementation of Pan Tompkins ECG QRS detector. 2014. 10.13140/RG.2.2.14202.59841

[26] Dasheiff RM , Kofke WA . Primarily generalized seizures are more effective than partial seizures in arousing patients from sleep. Neurol Res. 2003;25:63–67. 10.1179/016164103101200941 12564128

[27] Krone L , Frase L , Piosczyk H , Selhausen P , Zittel S , Jahn F , et al. Top‐down control of arousal and sleep: fundamentals and clinical implications. Sleep Med Rev. 2017;31:17–24. 10.1016/j.smrv.2015.12.005 26883160

[28] Moruzzi G , Magoun HW . Brain stem reticular formation and activation of the EEG. Electroencephalogr Clin Neurophysiol. 1949;1:455–473. 10.1016/0013-4694(49)90219-9 18421835

[29] Bernard C , Frauscher B , Gelinas J , Timofeev I . Sleep, oscillations, and epilepsy. Epilepsia. 2023;00:1–10. 10.1111/epi.17664 PMC10674035 37226640

[30] Saper CB , Chou TC , Scammell TE . The sleep switch: hypothalamic control of sleep and wakefulness. Trends Neurosci. 2001;24:726–731. 10.1016/S0166-2236(00)02002-6 11718878

[31] Blanco‐Centurion C , Gerashchenko D , Shiromani PJ . Effects of saporin‐induced lesions of three arousal populations on daily levels of sleep and wake. J Neurosci. 2007;27:14041–14048. 10.1523/JNEUROSCI.3217-07.2007 PMC2975593 18094243

[32] During MJ , Spencer DD . Extracellular hippocampal glutamate and spontaneous seizure in the conscious human brain. Lancet. 1993;341(8861):1607–1610. 10.1016/0140-6736(93)90754-5 8099987

[33] Steriade M , Amzica F . Slow sleep oscillation, rhythmic K‐complexes, and their paroxysmal developments. J Sleep Res. 1998;7:30–35. 10.1046/j.1365-2869.7.s1.4.x 9682191

[34] Parrino L , Halasz P , Tassinari CA , Terzano MG . CAP, epilepsy and motor events during sleep: the unifying role of arousal. Sleep Med Rev. 2006;10:267–285. 10.1016/j.smrv.2005.12.004 16809057

[35] Scott RC , Menendez de la Prida L , Mahoney JM , Kobow K , Sankar R , de Curtis M . WONOEP APPRAISAL: the many facets of epilepsy networks. Epilepsia. 2018;59:1475–1483. 10.1111/epi.14503 30009398

[36] Wang YL , Avigdor T , Hannan S , Abdallah C , Dubeau F , Peter‐Derex L , et al. Intracerebral dynamics of sleep arousals: a combined scalp–intracranial EEG study. J Neurosci. 2024;44:e0617232024. 10.1523/JNEUROSCI.0617-23.2024 PMC11026366 38471781

[37] Sforza E , Jouny C , Ibanez V . Cardiac activation during arousal in humans: further evidence for hierarchy in the arousal response. Clin Neurophysiol. 2000;111:1611–1619. 10.1016/s1388-2457(00)00363-1 10964073

[38] Azarbarzin A , Ostrowski M , Hanly P , Younes M . Relationship between arousal intensity and heart rate response to arousal. Sleep. 2014;37:645–653. 10.5665/sleep.3560 PMC4044744 24899756

[39] Eggleston KS , Olin BD , Fisher RS . Ictal tachycardia: the head‐heart connection. Seizure. 2015;23:496–505. 10.1016/j.seizure.2014.02.012 24698385

[40] Tao JX , Baldwin M , Ray A , Hawes‐Ebersole S , Ebersole JS . The impact of cerebral source area and synchrony on recording scalp electroencephalography ictal patterns. Epilepsia. 2007;48:2167–2176. 10.1111/j.1528-1167.2007.01224.x 17662060

[41] Huber R , Mäki H , Rosanova M , Casarotto S , Canali P , Casali AG , et al. Human cortical excitability increases with time awake. Cereb Cortex. 2013;23:1–7. 10.1093/cercor/bhs014 PMC3539451 22314045

[42] Lamberts RJ , Thijs RD , Laffan A , Langan Y , Sander JW . Sudden unexpected death in epilepsy: people with nocturnal seizures may be at highest risk. Epilepsia. 2012;53:253–257. 10.1111/j.1528-1167.2011.03360.x 22192074

[43] Kwon CS , Rafati A , Ottman R , Christensen J , Kanner AM , Jetté N , et al. Psychiatric comorbidities in persons with epilepsy compared with persons without epilepsy: a systematic review and meta‐analysis. JAMA Neurol. 2025;82:72–84. 10.1001/jamaneurol.2024.3976 PMC11589854 39585664

[44] Leng Y , Knutson K , Carnethon MR , Yaffe K . Association between sleep quantity and quality in early adulthood with cognitive function in midlife. Neurology. 2024;102:e208056. 10.1212/WNL.0000000000208056 PMC10870739 38170947

[45] Lee S , Kim JH , Chung JH . The association between sleep quality and quality of life: a population‐based study. Sleep Med. 2021;84:121–126. 10.1016/j.sleep.2021.05.022 34147026

[46] Strzelczyk A , Aledo‐Serrano A , Coppola A , Didelot A , Bates E , Sainz‐Fuertes R , et al. The impact of epilepsy on quality of life: findings from a European survey. Epilepsy Behav. 2023;142:109179. 10.1016/j.yebeh.2023.109179 37058861

