# Supplementary material for: Epileptic arousals: A neglected clinical entity
Source: Epilepsia. 2026 Aug 19;67(11):5781–96. doi: 10.1002/epi.70442 (PMC13768902; doi:10.1002/epi.70442)
Supplement: Supplementary file 1 [file EPI-67-5781-s001.pdf]

Supplementary Materials & Methods: Extended Definition of Arousal Types

- Fast Arousal (equivalent to CAP A3): Abrupt shift in EEG frequency to fast-rhythm low-voltage including alpha, theta, or frequencies above 16 Hz (excluding spindles). EEG desynchronization > 50%.
- Intermediate Arousal (equivalent to CAP A2): Abrupt shift in EEG frequency to mixed fast- and slow-rhythm, variable-voltage including polyphasic EEG bursts. EEG desynchronization between 20% and 50%.
- Slow Arousal (equivalent to CAP A1): Abrupt shift in EEG frequency to slow-rhythm high-voltage including K-complexes, delta bursts, and vertex sharp transients. EEG desynchronization < 20%.

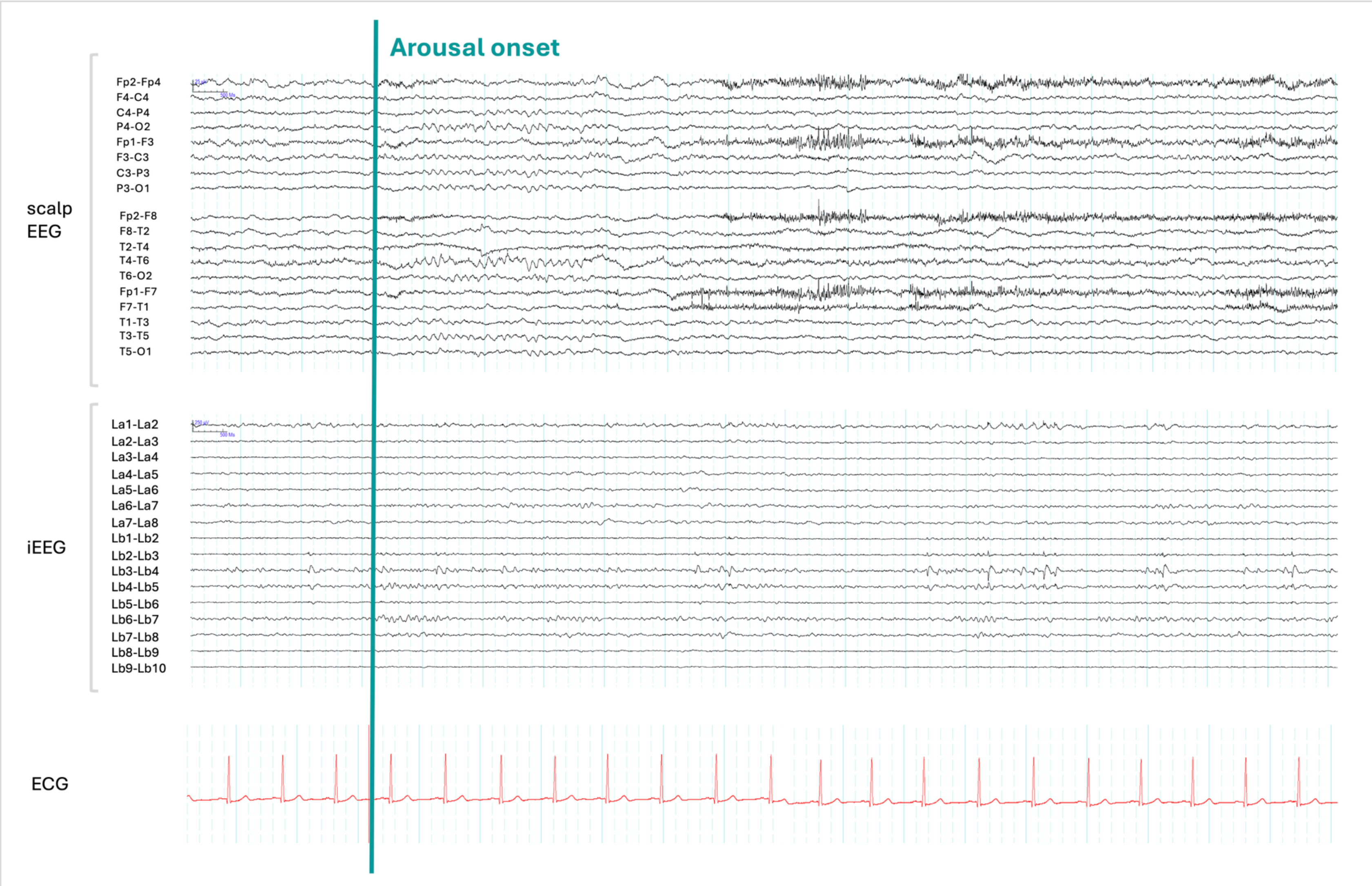

Supplementary Fig. S1: Physiological arousal in simultaneous intracranial and scalp EEG.
A fast-type arousal during N2 sleep becomes apparent on scalp EEG (vertical line). No ictal activity is detectable in either intracranial or scalp EEG. *EEG: Electroencephalography, iEEG: Intracranial EEG, ECG: Electrocardiography*

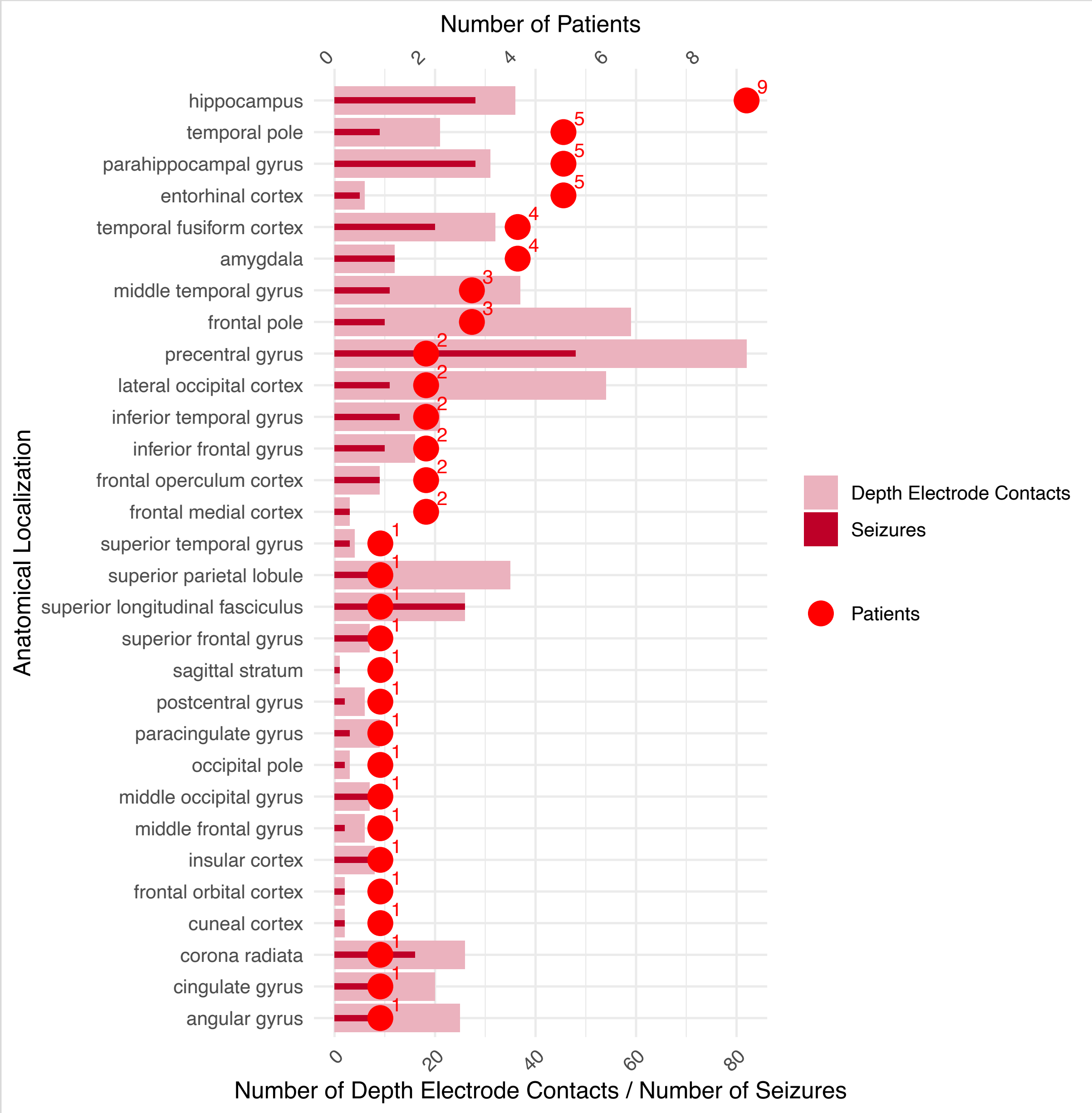

Supplementary Fig. S2: Seizure onset zones in epileptic arousals. Visually identified ictal activity at seizure onset in iEEG channels mapped to associated anatomical localization. For each anatomical localization, number of depth electrode contacts (thick pink bar), seizures (thin dark red bar) and patients (red points) were counted. 30 anatomical regions were identified as part of the seizure onset zone. *iEEG: Intracranial electroencephalography.*

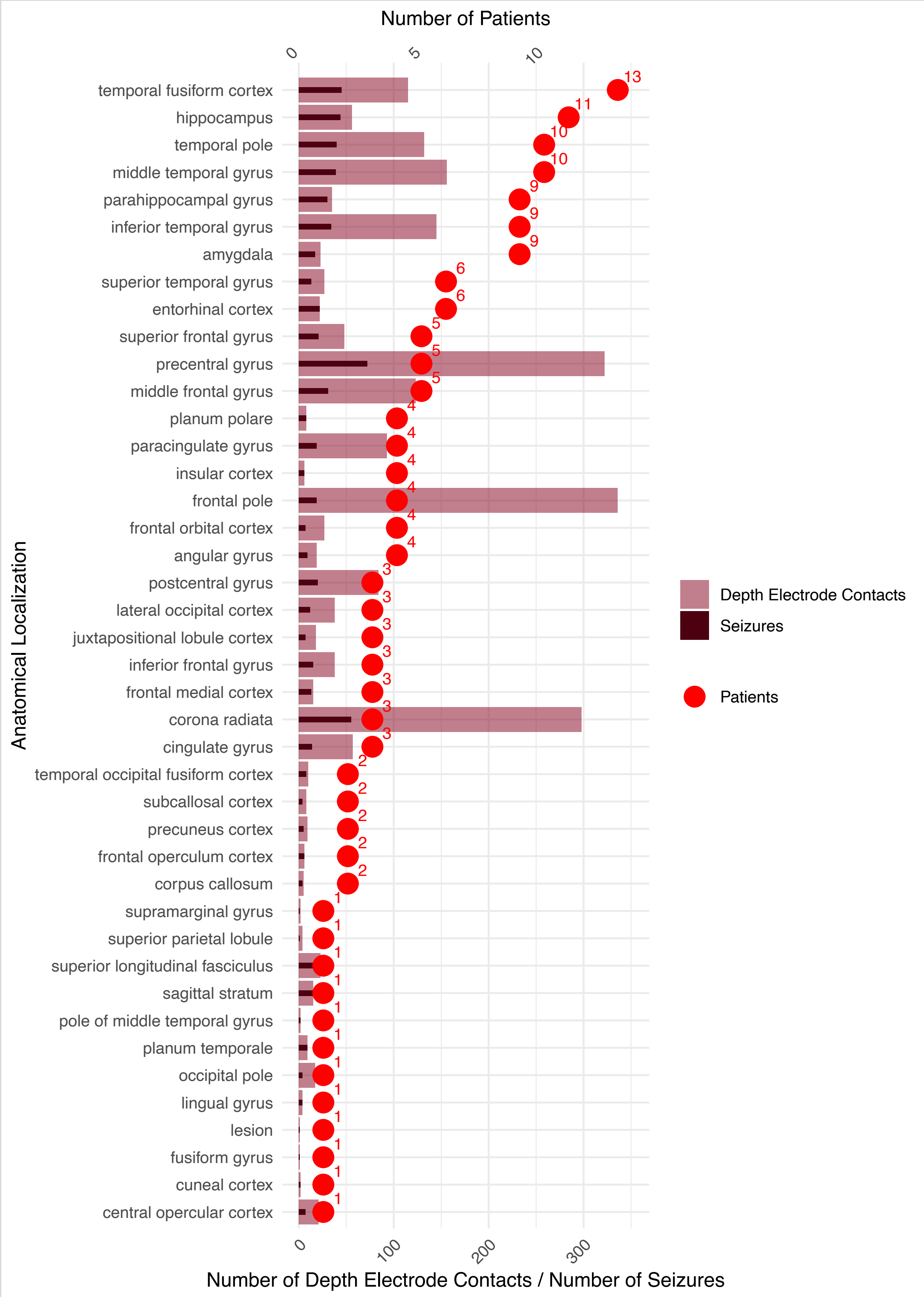

Supplementary Fig. S3: Maximum seizure propagation zones in epileptic arousals. Visually identified ictal activity at maximum extent of seizure propagation in iEEG channels mapped to associated anatomical localization. For each anatomical localization, number of depth electrode contacts (think dark red bar), seizures (thin black bar) and patients (red points) were counted. *iEEG: Intracranial electroencephalography.*

Supplementary Table S1: Influence of Epileptic Arousals and Physiological Arousals on Heart Rate

**A. HR and HR increase (Wilcoxon rank sum test)**						
**phase**	**EA** *median (IQR)*	**PA** *median (IQR)*	**Statistic W** (EA vs. PA)	**p**		
Pre-arousal	0.000 (0.025)	0.000 (0.02)	25,933,260	0.958		
Post-arousal	0.074 (0.156)	0.027 (0.083)	31,153,661	< 0.001		
pre vs. post	W = 40,239,197	W = 38,044,526	-	< 0.001/ < 0.001		
**B. Distribution of HR Modality (Hartigan's dip test)**						
	**EA**		**PA**			
**Time window**	**Statistic D**	**p**	**Statistic D**	**p**		
0−48s[a]	0.005	0.292	0.003	0.911		
25−48s[a]	0.016	< 0.001	0.005	0.765		
**C. EA vs. PA: Variability of HR (IQR) and Classification Performance**						
**Time window**	**Permutation p**	**ROC-AUC [95% CI]**	**Sens** *(%)*	**Spec** *(%)*	**Acc** *(%)*	**Optimal Cutoff** *(Youden Index)*
0−48s[a]	0.022	0.746 [0.714, 0.777]	50	92	71	0.070
25−48s[a]	0.007	0.995 [0.991, 0.998]	98	94	96	0.094

HR: Heart rate, EA: Epileptic arousal, PA: Physiological arousal, IQR: Interquartile range, s: second, ROC: Receiver operating characteristic, AUC: Area under the curve, CI: Confidence interval, Sens: Sensitivity, Spec: Specificity, Acc: Accuracy. [a] post-arousal.
